# Multi-channel microfluidic chip coupling with mass spectrometry for simultaneous electro-sprays and extraction

**DOI:** 10.1038/s41598-017-17764-6

**Published:** 2017-12-12

**Authors:** Cilong Yu, Fei Tang, Xiang Qian, Yan Chen, Quan Yu, Kai Ni, Xiaohao Wang

**Affiliations:** 1 College of Mechatronics and Control Engineering, Shenzhen University, Shenzhen, 518060 China; 2 Division of Advanced Manufacturing, Graduate School at Shenzhen, Tsinghua University, Shenzhen, 518055 China; 3 Shenzhen Institutes of Advanced Technology, Chinese Academy of Sciences, Shenzhen, 518055 China; 4 The State Key Laboratory of Precision Measurement Technology and Instruments, Department of Precision Instrument, Tsinghua University, Beijing, 100084 China

## Abstract

Considering the advantages and research status of microfluidic chip coupling with mass spectrometry (MS), a microfluidic chip-based multi-channel ionization (MCMCI) for the extraction of untreated compounds in complex matrices without sample pretreatments was developed. Quantitative analysis of human urine spiked with various rhodamine B concentrations was also performed, and good linearity was obtained. Comparing to the macro ionization device, MCMCI significantly improved the integration of ionization source, simplified the operation of such a device, and greatly increased the signal intensity with much lower gas pressure. Comparison of our MCMCI with two and three gas channels indicated that the liquid–liquid extraction process before spraying and after spraying produced similar MS results. Moreover, this MCMCI with three gas channels also implemented simultaneous dual sprays with high DC voltages, the interference of two samples was minor and ion suppression effect was drastically alleviated. Such advantages may easily enable internal calibration for accurate mass measurement. Furthermore, dual extraction can be implemented by integrating such multi-spray configuration, which can improve the extracted signal intensity and sensitivity. These technologies open up new avenues for the application of microfluidic chip coupling with MS.

## Introduction

Microfluidic chips have been widely applied in various research fields due to their efficient and fast separations, integrating complex sample pretreatment functions and automatic manipulation of small sample volumes^1–5^. The coupling of microfluidic chips with MS has gained considerable attention in the MS community^6–8^. However, most microfluidic chip-based ionizations only possess one liquid channel for spraying, lacking gas atomizing, desorption function, and many other ionization methods^9^. Studies on MCMCI are even less^10^, let alone the studies on extractive electrospray ionization (EESI) and internal calibration in microfluidic chips^11,12^.

Among the current atmospheric pressure ionization techniques^13^, dual-channel ionization has been widely applied for EESI^14–17^, internal calibration^12,18,19^, and protein analysis^20–22^. EESI plays a significant role in MS community^15^, which was similar to the FD-ESI proposed by Shiea *et al*.^23^. Based on the theory of liquid–liquid extraction among the colliding micro-droplets from two different spray sources, EESI owns the capability to directly analyze compounds in the solution, gas phase, and other complicated matrices without sample pretreatments^14,24^. However, optimizing the angle between the two sprayers, which significantly affect the sensitivity and stability of the measurements is difficult^25^. Despite that the air-flow-assisted EESI ionization source^25^ was developed to overcome this disadvantage, the complex macrostructure was still difficult to operate and optimize. Comparatively, microfluidic chip-based ionization is compact and integrated, making it particularly facile for coupling with MS and even with portable MS in the future. Lu *et al*.^11^. proposed a dual-channel electrospray microchip for solutions to mix at the spray tip, where high voltage was applied on the aqueous sample rather than the organic buffer solution. The relative standard deviation (RSD) of the total ion current (TIC) obtained from mixed spray was rather high.

In addition, dual-channel ionization is an effective approach to realize internal calibration for accurate mass measurements because the dual channels might prevent ion suppression of the two compounds^12,18,19^. However, numerous methods reported with two independent ESI emitters failed to introduce the analyte and reference solutions into a single mass spectrometry inlet simultaneously because the interference among the electric fields of the adjacent sprayers is severe^12,26–28^. By extending the ion delivery distance^29,30^ or applying the AC power^18^, both of them implemented simultaneous dual-channel sprays. However, the macrostructure was complicated and the AC power achieved half-lower signal intensity than the DC power. Ramsey *et al*.^12^ reported a microfluidic chip with dual ESI emitters for internal calibration. The chip sprayed two compounds sequentially by dynamic control of the ESI voltages.

In our previous work^1,9^, a microfluidic dual-channel self-aspiration sonic spray ionization chip was reported. This device enabled dual channels to spray simultaneously without high voltage, but the signal intensity and stability must be further improved. Herein, we report a novel design of MCMCI with high voltage for MS. This MCMCI enabled the extractive solvent to extract samples with the assistance of airflow. Besides, another similar microfluidic chip structure with high DC powers applying on the dual channels implemented to spray two samples simultaneously, which successfully prevented the ion suppression to some extent. These advantages simplified the operation of microfluidic chip-based ionization coupling with MS because optimizing the angle of EESI^25^, positioning the dual emitters at the MS inlet^28^, and dynamic controlling the ESI voltages for dual sprays were necessary^12,31^. Moreover, dual extraction (dual EESI) was realized based on the multi-spray configuration with high DC powers, which can improve the extracted signal intensity and sensitivity. All of these advantages of MCMCI were particularly suitable for coupling with portable MS in the future and may drastically extend the application of microfluidic chip in MS community; such applications are throughput increments^32^, sensitivity improvements^33^, and rapid mixing for ion/ion reactions^21,22,34^.

## Results

### MCMCI with two gas channels for extraction

**Figure 1:**
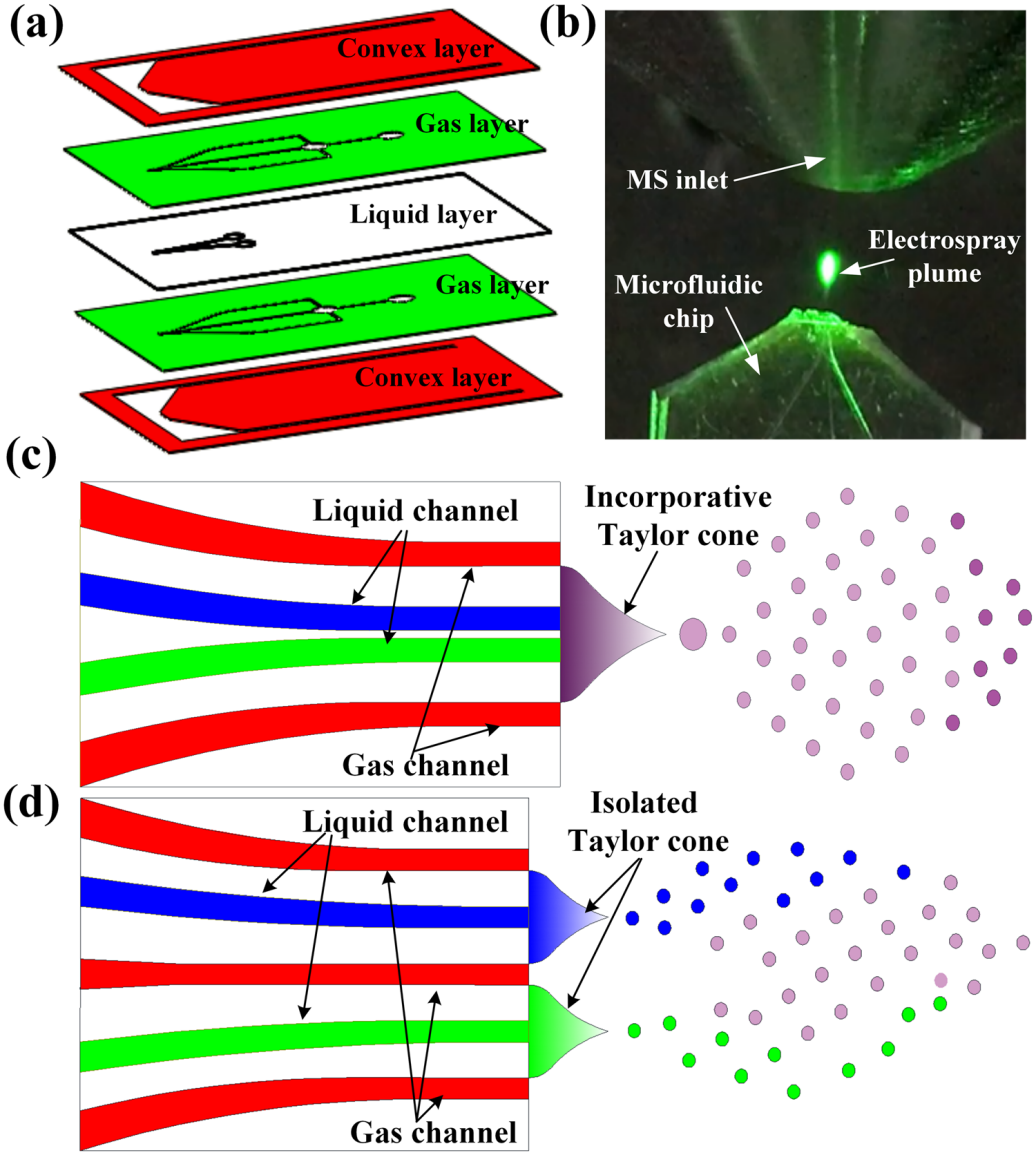
(**a**) Five layers of MCMCI. (From top to bottom: convex layer, gas channel layer, liquid channel layer, gas channel layer, convex layer). (**b**) The coupling of MCMCI and MS. The electrospray plume was illuminated with green diode lasers when the dual channels sprayed simultaneously. (**c**) The structure of MCMCI with two gas channels with samples spraying. (**d**) The structure of MCMCI with three gas channels with samples spraying.

**Figure 2:**
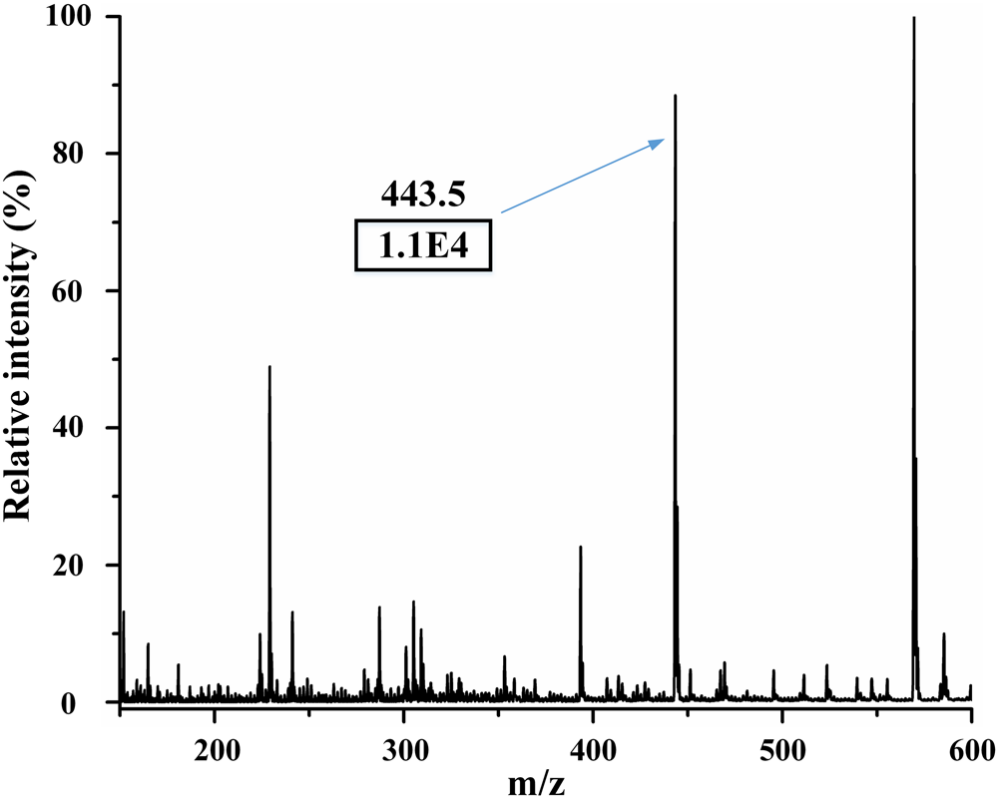
MS spectrum of undiluted human urine spiked with 1 μM Rhodamine B. The MCMCI with two gas channels was utilized.

The five layers design of microfluidic chip ionization source is presented in Fig. 1 and the whole schematic of four microfluidic chips is shown in Figure S1, ESI†. For MCMCI with two gas channels (the distance between two liquid channels was 10 µm), the two liquid channels were close to each other without a separating gas channel, and the liquids rapidly mixed at the tip of the device as one Taylor cone prior to spraying, which is shown in Fig. 1c. This result was similar to the study of Michel Prudent *et al*.^10^, except for the bilateral gas channels. Thus, the extraction mechanism of MCMCI with such configuration may be mainly based on the liquid–liquid extraction prior to spraying (*i.e*., the tip-mixing EESI mode). To demonstrate that this technique was applicable to detect complex matrices without sample preparation steps, undiluted human urine with 1 µM rhodamine B was tested. Figure 2 shows the MS spectrum of the urine test. The intensity and signal to noise ratio of rhodamine B were very high. For comparison, we also performed the experiment of 1 μM Rhodamine B in methanol and water sprayed by the commercial electrospray ionization with 5 KV high voltage as shown in Figure S2 ESI†. The signal intensity was similar with our on-chip extraction using real biological samples with same conditions, which clearly demonstrate the effeteness of our on-chip configurations.

**Figure 3:**
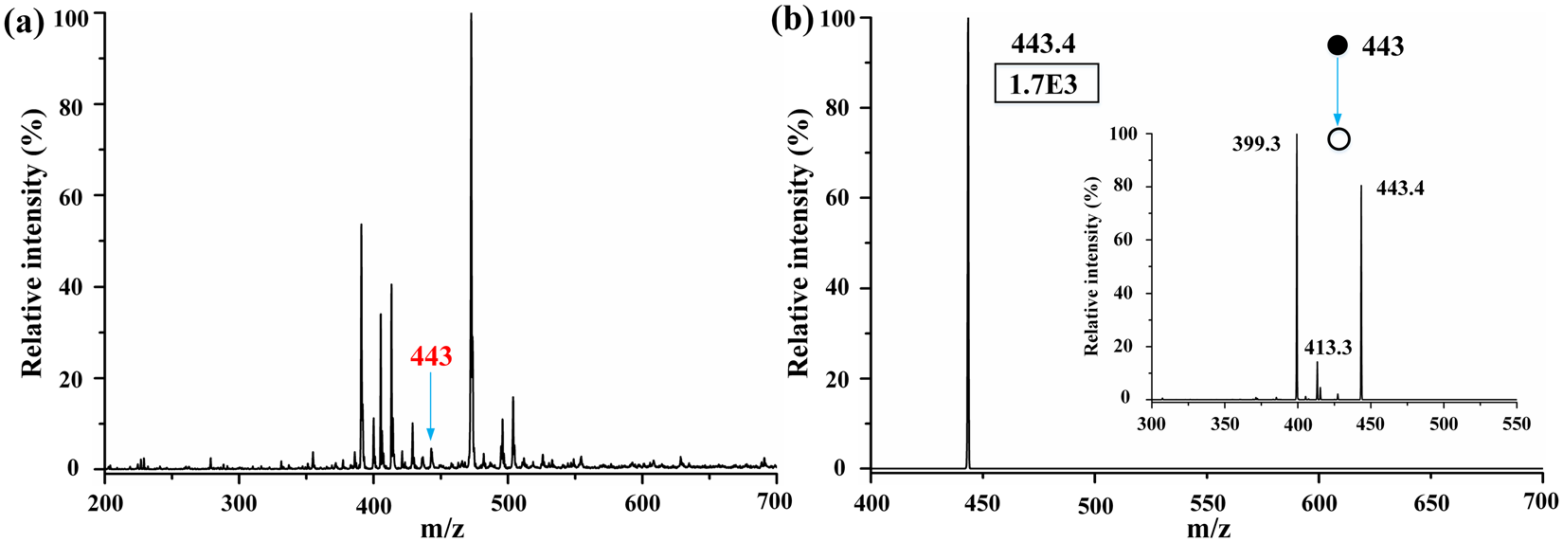
(**a**) MS spectrum of human urine (diluted five times by purified water) spiked with 0.01 µM rhodamine B. (**b**) Isolation MS spectrum of 443.4 in MS^2^ (parent mass 443 with width 1) without any collision energy, the signal intensity of 443.4 was approximately 1.7E3. The insert shows the MS^2^ spectrum of m/z 443.4 with collision energy.

Figure 3 describes the MS signal of rhodamine B in human urine at low levels. Although the background intensity was high in the full scan shown in Fig. 3a, the rhodamine B intensity shown in Fig. 3b was high enough for analyzing the sample when MS^2^ was utilized, which convincingly proved that this microfluidic chip possessed the ability to detect low concentration samples. Moreover, to test the stability of this microfluidic chip-based EESI, an experiment of purified water spiked with 6 µM rhodamine B was performed over 10 min (Figure S3, ESI†). The RSD of TIC in this experiment was only 2.3%. These experiments demonstrated that the sensitivity and stability of MCMCI were excellent.

**Figure 4:**
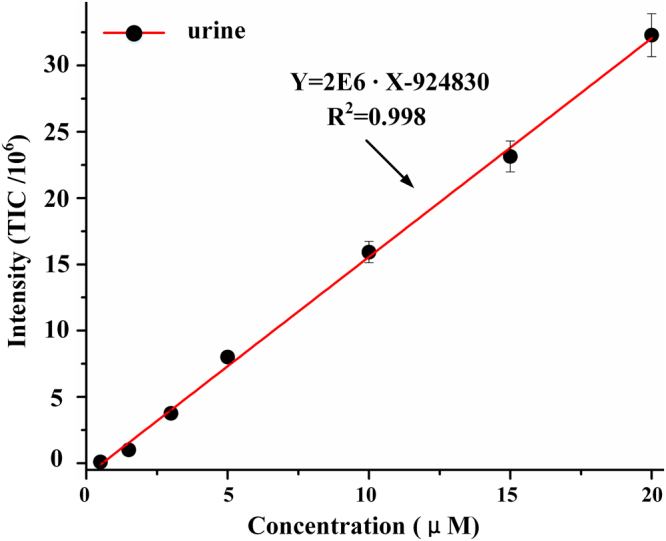
Quantitative analysis of human urine spiked with rhodamine B (0.5, 1.5, 3, 5, 10, 15, and 20 µM), separately. Human urine was diluted 5 times by purified water. (Each point was tested for three times and each time lasts for one minute. TIC mass range was from 443 to 444).

To further demonstrate the characteristics of this extraction function, experiments under the same extraction conditions with various rhodamine B concentrations were performed. The distance between MS inlet and microfluidic chip for experiments was approximately 6 mm. For such illustrative calibration experiments, we diluted the urine with water five times for the following EESI experiments because the urine collected once from one person was not enough, as different concentration solutions for quantitative analysis required large amounts of urine background solvent. If urine was collected from different people or different times, the urine solvent background might be various and affect the quantitative analysis. Figure 4 shows a plot of TIC intensity as a function of the original rhodamine B concentrations in human urine. Good linearity was obtained. Under such experimental conditions with LCQ MS, the current best linear range is approximately 0.5 µM to 20 µM, because the thermo LCQ mass spectrometer with three-dimensional ion trap owned the capacity limitation for high concentration quantitative analysis. For comparision, we also performed the experiment of 1 μM Rhodamine B in undiluted urine and urine diluted by five times sprayed by the commercial electrospray ionization with 5 KV high voltage respectively as show in Figure S4 ESI†. Firstly, the signal was similar no matter the urine was diluted or not; secondly, the signal intensity of Rhodamine B in urine was much worse than in methanol and water solution as shown in Figure S2 ESI†. Thus, we can speculate that the five times dilution had little influence in these experiments and the MCMCI did really improve the signal intensity greatly comparing to commercial ESI.

**Figure 5:**
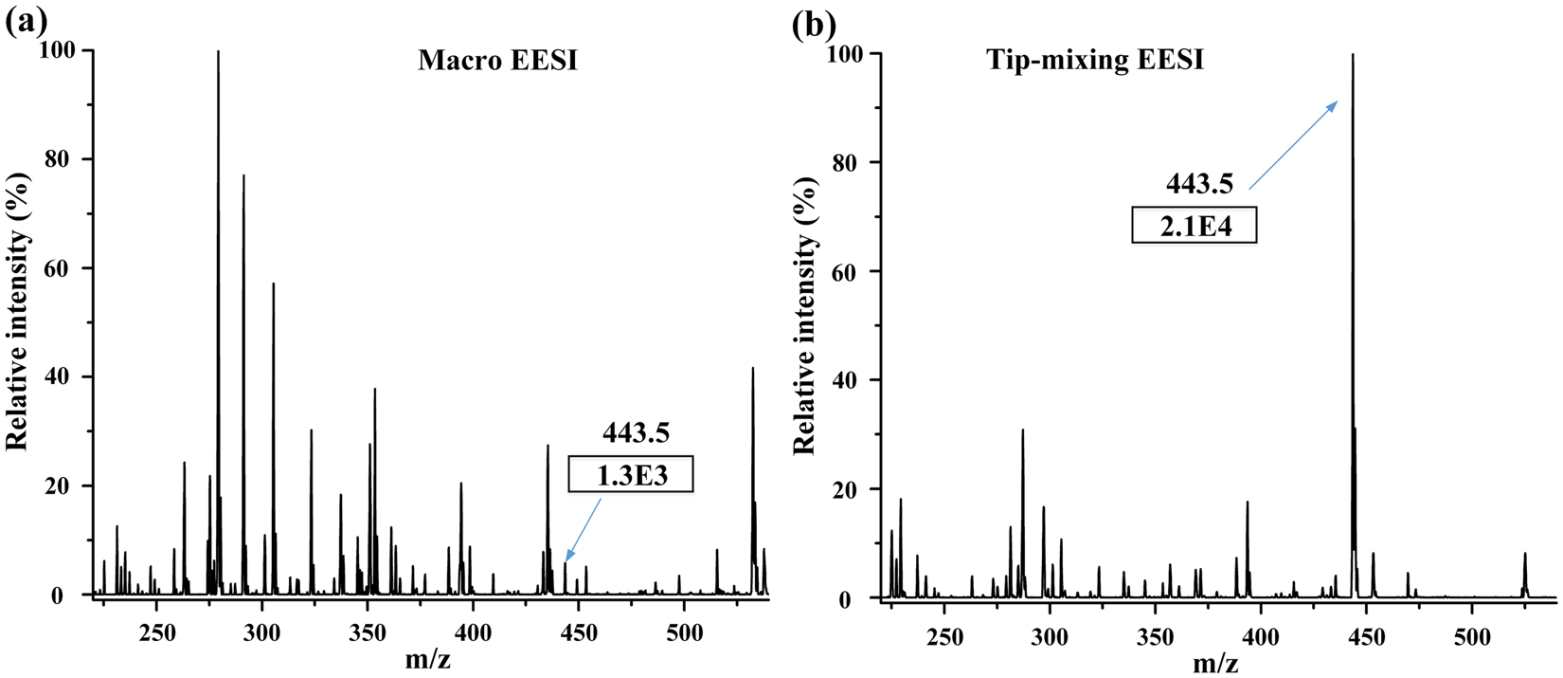
(**a**) MS spectrum of human urine (diluted 5 times by purified water) spiked with 3 μM rhodamine B measured by macro EESI. (**b**) MS spectrum of human urine (diluted 5 times by purified water) spiked with 3 μM rhodamine B measured by microfluidic chip tip-mixing EESI. The pressures on the liquid channels were all 300 mbar, while the pressure was 3.5 bar on the gas channel inlet, high voltage (5 kV) was applied on the extractive solvent.

The comparison experiments of this tip-mixing EESI and macro EESI were also conducted. The structure and position of macro EESI was based on the references^14,17^, the picture of which is shown in figure S5, ESI†. Two concentrations of rhodamine B in urine (0.1 and 3 µM) were tested by using the macro EESI and tip-mixing EESI. Each concentration was measured for six times, and each time lasted for 1 minute. The experimental conditions were the same for the macro EESI and tip-mixing EESI. The intensities of macro EESI were much lower than the tip-mixing EESI, particularly for high concentrations. The average extracted ion intensities (mass range from 443 to 444) of macro EESI (0.1 and 3 µM) were 3.04E3 and 3.62E3, respectively. However, the average extracted ion intensities (mass range from 443 to 444) of the tip-mixing EESI (0.1 and 3 µM) were 5.38E3 and 1.15E5 separately. Figure 5 shows the MS spectrum of human urine (diluted five times by purified water) spiked with 3 µM rhodamine B. The intensity and signal-to-noise ratio of macro EESI were much worse than the tip-mixing EESI. Considering that the high gas pressure (approximately 10 bar) was usually applied on the macro EESI, we also attempted to increase the gas pressure to improve the signal intensity. Thus, macro EESI was utilized to measure the signal of 15 µM rhodamine B in human urine with high gas pressure. When the experimental conditions were the same as mentioned above, the intensity of 15 µM rhodamine B was 5.42E3. When the gas pressures applied on the two sprayers increased to 10 bar, the signal intensity increased to 3.9E4, which was lower than the intensity of 3 µM from the tip-mixing EESI. Despite much higher gas pressure was applied on the macro EESI, the intensity of the macro EESI was still much lower than microfluidic chip.

### MCMCI with three gas channels for extraction and dual sprays with high DC powers

For MCMCI with three gas channels, a separating gas channel was placed between the two liquid channels; thus, the fluids did not mix at the tip of the device before they were sprayed; two liquids formed two isolated Taylor cones at each liquid channel front end, as shown in Fig. 1d. According to such observation and assumption that the two liquid channels in MCMCI with three gas channels served as two isolated electrospray ionizations, the samples were isolated until they were sprayed out. Therefore, the extraction mechanism of MCMCI with such configuration might be mainly based on the theory of liquid–liquid extraction among colliding droplets from two different spray sources (i.e., the droplet-collision EESI mode). The EESI mass spectrum signal of MCMCI with three gas channels is shown in Figure S6, ESI†; the signal intensity (9.7E4) was similar but a little lower than the signal of MCMCI with two gas channels (1.5E5), as shown in Figure S3b, ESI†. These results suggested that for MCMCI-based EESI, tip-mixing EESI based on the liquid–liquid extraction before spraying and droplet-collision EESI based on the liquid–liquid extraction among the colliding droplets after spraying shared very similar MS results.

**Figure 6:**
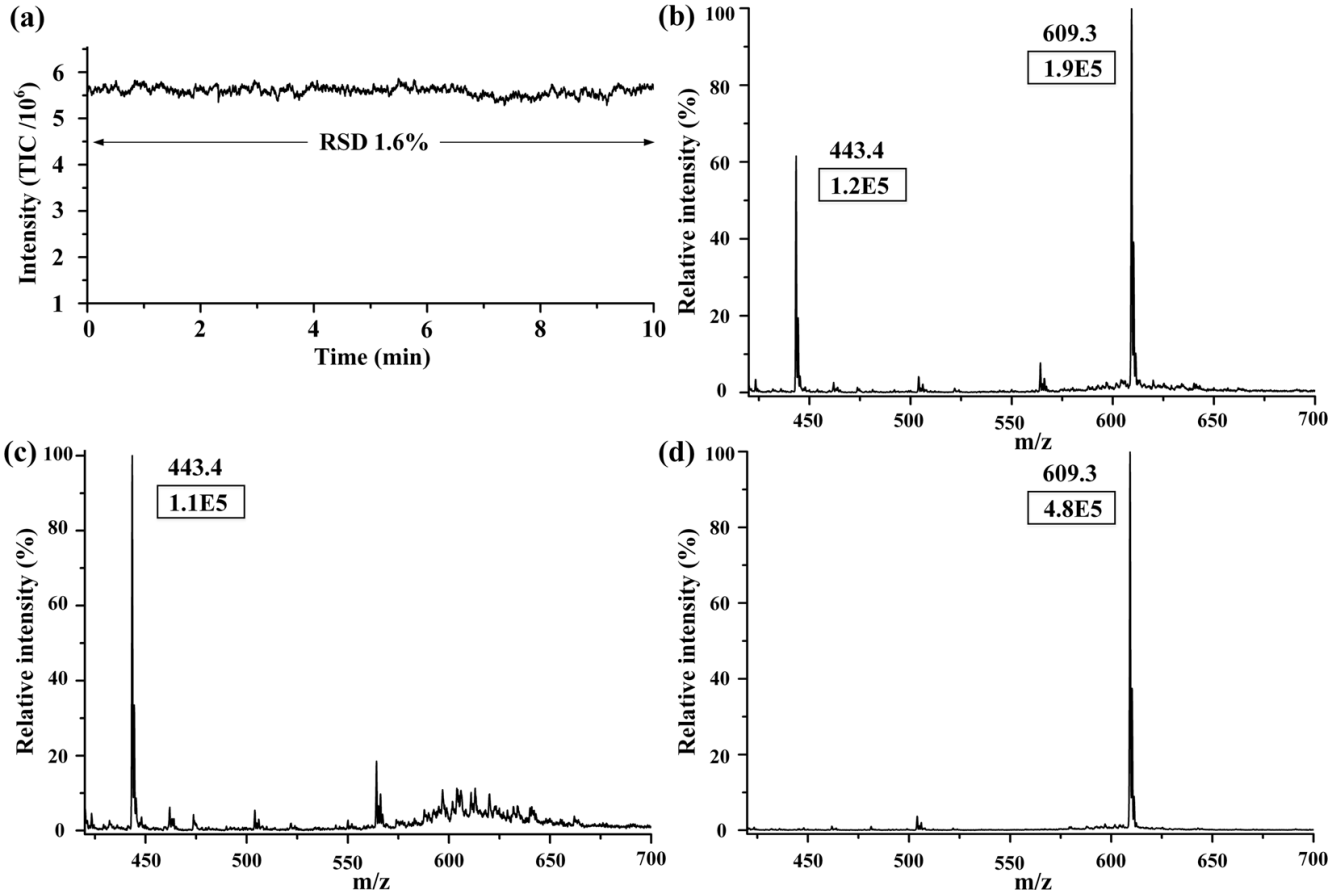
(**a**) Long-term stability of purified water spiked with 6 µM rhodamine B and the mixture solution of 20 µM arginine, 20 µM ciprofloxacin, and 16.4 µM reserpine with 1% acid in water and methanol (1:1,v/v). (**b**) MS spectra of 6 μM Rhodamine B and 16.4 μM reserpine when dual channels were used. The signal intensity of Rhodamine B and reserpine was 1.2E5 and 1.9E5, respectively. (**c**) MS spectrum of 6 μM Rhodamine B when only Rhodamine B channel was utilized. The signal intensity of Rhodamine B was 1.1E5. (**d**) MS spectrum of 16.4 μM reserpine when only reserpine channel was utilized. The signal intensity of reserpine was 4.8E5. Rhodamine B and reserpine were in water and methanol (1:1,v/v).

Based on the isolated spraying assumption, the interferences and ion suppression effect between the two liquid channels might be relieved with high DC powers supplied simultaneously. Figure 6a shows the long-term stability of the two samples spraying at such situation. RSD was only 1.6%, which definitely proved that the MCMCI could spray stably. Moreover, comparing the signal intensities of dual emitters (Fig. 6b) with single emitter (Fig. 6c and d), the signal for reserpine was decreased by a factor of 2.5, whereas rhodamine B remained at similar signal intensity. This finding proved that the interference between two sprays existed but was not severe, as pointed out in the previous study^18^ where the signal intensity decreased by a factor of 4.7. Thus, our MCMCI avoided the severe interference among the DC electric fields of the adjacent sprayers, which existed in the previous reported devices^12,18^. Thus, AC power was utilized in the previous study^18^, but the signal intensity of which was nearly half of the DC nanoESI because of the half cycle’s negative voltage in AC power; furthermore, optimizing the amplitude and waveform of AC power is also complicated^18^.

**Figure 7:**
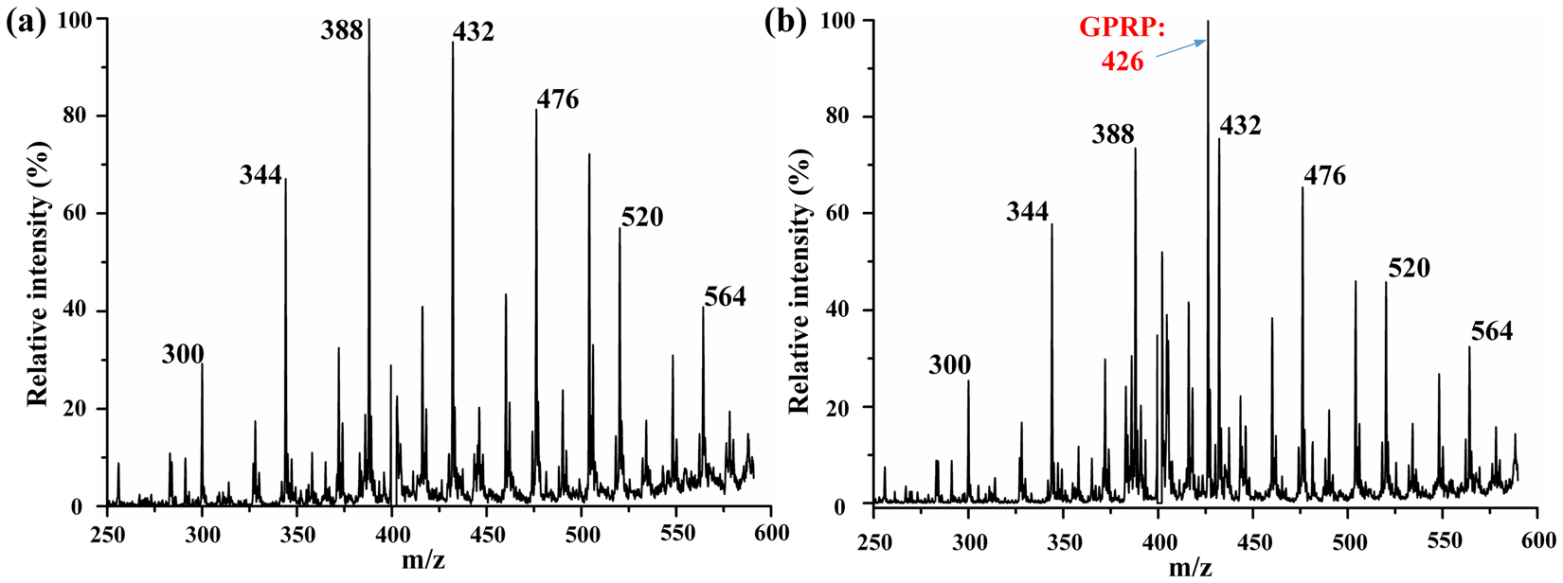
(**a**) MS spectrum of mixed solution peptide GPRP and PEG-400 sprayed by one channel. Both samples were all approximately 58.5 µM in methanol and water (1:1,v/v). (**b**) MS spectrum of peptide GPRP and PEG-400 sprayed by two channels separately. Both samples were all approximately 117 µM in methanol and water (1:1,v/v).

To further illustrate the ion suppression effect, peptide GPRP and PEG-400 were utilized in the experiments. Under the same experimental conditions, the MS spectra of peptide GPRP and PEG-400 is illustrated in Fig. 7. The signal intensity of peptide GPRP was submerged when the mixture of PEG-400 and peptide was sprayed by one channel, ion suppression effect existed severely, as shown in Fig. 7a and proved in the previous study^18^. While the PEG-400 and peptide were sprayed separately in two channels, it could easily obtain comparative signals of PEG-400 and the peptide simultaneously, as demonstrated in Fig. 7b. These results further proved the assumption that samples were isolated until they were sprayed out, and the extraction was occurred after spraying in the MCMCI with three gas channels. Furthermore, such dual spraying with high DC powers supplied simultaneously may have two advantages. First, such configuration is suitable for the application of accurate mass measurements by internal calibration. Second, such configuration may be extended to integrate dual extraction function with simultaneously supplied high voltages on the multi-spray channels (the extraction solvent channels were set at both side of the sample channel), as shown in the following section.

### Microfluidic Chip Ionization source for Dual EESI

**Figure 8:**
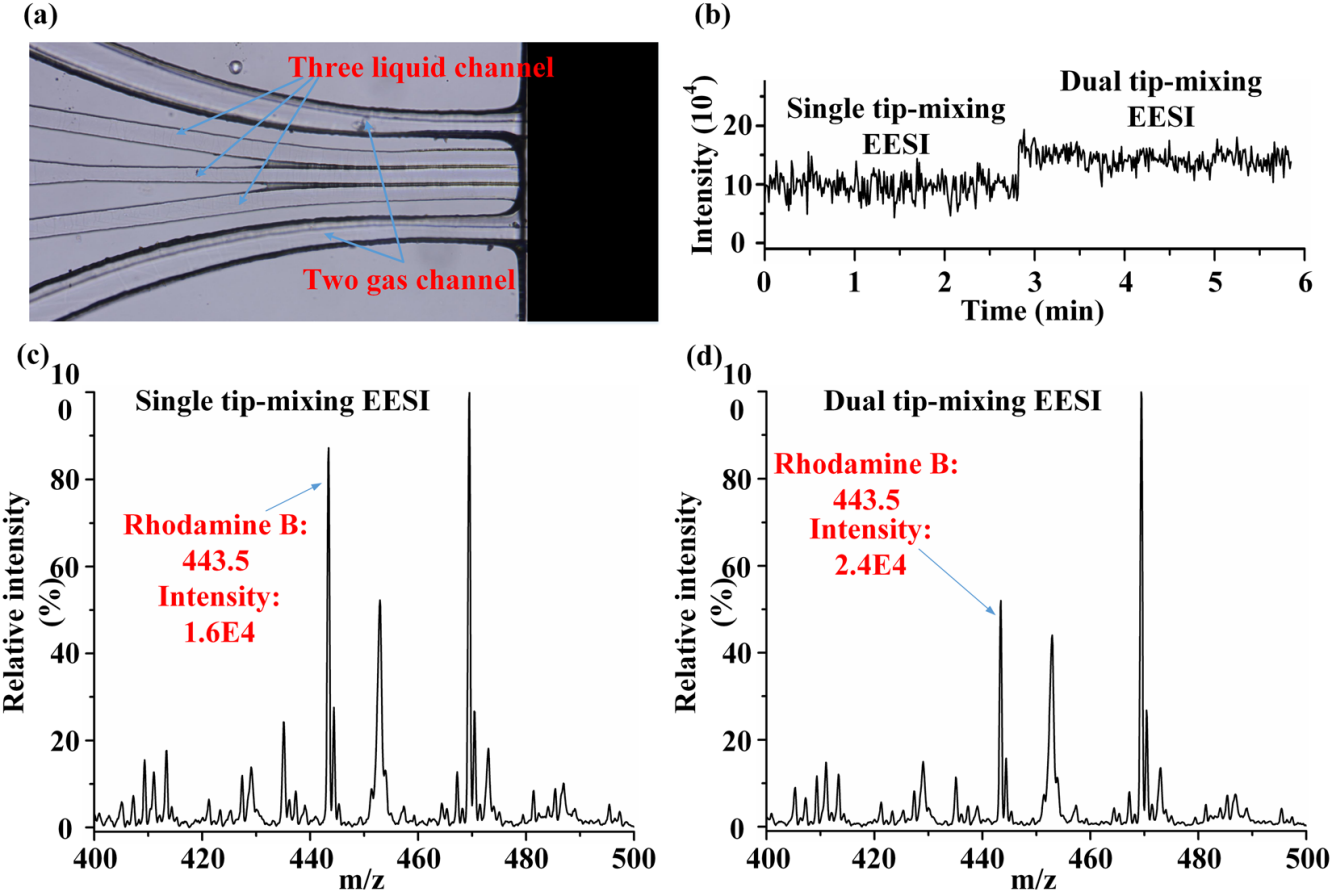
(**a**) Structure of MCMCI with two gas channels and three liquid channels for dual tip-mixing EESI. (**b**) TIC intensity of 0.1 µM rhodamine B (extracted mass range from 443 to 444). In the first three min, only one extracted channel sprayed with sample channel (i.e., the single tip-mixing EESI); in the last three min, two extracted channels sprayed with sample channel (i.e., the dual tip-mixing EESI). (**c**) MS spectrum of 0.1 µM rhodamine B in the single tip-mixing EESI mode. (**d**) MS spectrum of 0.1 µM rhodamine B in the dual tip-mixing EESI mode.

Microfluidic chip ionization for dual EESI was implemented by integrating the functions of extraction and multi-spray configuration with the above-mentioned high DC powers. Multi-sprays mean three liquid spraying channels were set with two (dual tip-mixing EESI) or four (dual droplet-collision EESI) gas channels, as illustrated in Figures S1c and S1d, respectively. Figure 8a shows the structure of dual tip-mixing EESI. The sample was introduced through the middle liquid channel and the two extractive solvents distributed at both sides of the sample, these three liquids also fused together as one Taylor cone at the tip of the liquid channels. The TIC intensity of 0.1 µM rhodamine B is described in Fig. 8b. Dual tip-mixing EESI significantly improved the extraction sensitivity and signal intensity, which can also be clearly examined in Fig. 8c and d. This dual EESI helps improve the signal intensity, but a larger Taylor cone might cause a slight instability of spraying compared with a smaller one. Dual droplet-collision EESI with three liquid channels and four gas channels was also designed. The experiments were also performed; Figure S7, ESI† also proved that dual droplet-collision EESI could obtain higher signal intensity than single EESI.

## Discussion

EESI was implemented in microfluidic chip coupling with MS for the first time. Stable extraction function enabled quantitative analysis of untreated compounds in complex matrices, and the good linearity (R^2^ = 0.998) showing in Fig. 4 proved that metabolites in biological samples might be quantitatively analyzed without any pretreatments by MCMCI coupling with MS. More importantly, compared with the macro EESI device, microfluidic chip ionization reflects enormous superiority. As demonstrated in Fig. 5, microfluidic chip based tip-mixing extraction obtained higher signal intensity, sensitivity and signal to noise ratio with much lower gas pressure; moreover, optimizing the angle between two sprayers was elided, which significantly simplified the operation of EESI device. MCMCI with three gas channels also enabled extraction in the droplet-collision mode. The mass spectrum of tip-mixing and droplet-collision extraction was similar as shown in Figure S3b and S6, ESI†. In other words, the MCMCI with two and three gas channels both possessed the advantages of microfluidic chip extraction.

Owing to the electric interference between two adjacent sprayers, dual simultaneous electrosprays based on microfluidic chip was barely reported. This study demonstrated the function of spraying two samples simultaneously with high DC voltages. The long-term stability of two samples spraying simutaneously proved that the MCMCI could spray stably, as shown in Fig. 6a, where the RSD was only 1.6%. The alleviation of ion suppression effect made it suitable for internal calibration for accurate mass measurement. MCMCI was free from interference of electric field, it might benefit from the dual spray channels rather than the dual spray tips, which generated two high electric fields.

Dual extraction microfluidic chip ionization was also developed by integrating the functions of extraction and dual simultaneous electrosprays. Such multi-spray configurations were beneficial to further improve signal intensity and sensitivity. This fully embodied the integration characteristics of microfluidic chip.

In summary, compared with the macro ionization device, tip-mixing and droplet-collision extraction based on the microfluidic technology significantly improved the integration of ionization and simplified the operation of such device, and the dual extraction configuration can further improve the performance, making it particularly suitable for coupling with portable MS in the future. This paper mainly introduced the new chip structure, realized the fabrication process and verified its abilities; further application will be conducted in our future works, which we will consider blood samples, sweat samples and oil samples with different analytes, besides the urine samples. These advantages may significantly extend the application of microfluidic chip in MS community; such applications are throughput increments^32^, sensitivity improvements^33^, and protein analysis^34^.

## Methods

### Materials and Equipment

HPLC-grade methanol and acetic acid were purchased from Merck KGaA (Darmstadt, Germany). PDMS elastomer base and curing agent (Sylgard 184) were purchased from Dow Corning (Midland, MI, USA). SU-8 photoresist was obtained from Microchem Co. (Naton, MA, USA). Samples used in this study were obtained from commercial sources. The urine was collected from one adult directly. All solutions were supplied to the microfluidic chip through short stainless steel tubes embedded in the reservoirs using a pneumatic pressure controller (MFCS, Fluigent, Paris, France). The high voltage generated by a power supply module (Dongwen High Voltage Power Supply Co., Ltd, Tianjin, China) was applied on the stainless steel tube of the extractive solvent. A thermo LCQ MS(Thermo Fisher Scientific Inc., Waltham, MA, SA) was coupled to the microfluidic chip, and MS data were collected by the computer. The detailed schematic of the platform was shown in Figure S8, ESI†. The MCMCI was held by a laboratory-built platform and coupled to the ion trap mass spectrometer. The distance between the microfluidic chip emitter and the MS inlet orifice was adjusted by a xyz-manipulator.

### Design of MCMCI

The three layers of MCMCI were first designed by using AutoCAD (Autodesk, Inc., San Rafael, CA, USA). Then, the three photo masks were manufactured by Qingyi Precision Mask Making Co., Ltd (Shenzhen, China). The whole schematic of microfluidic chips is shown in Figure S1, ESI†. The width was 30 µm at the end of all channels. The figure contains a so-called tip-mixing extraction configuration with two gas channels (Figure S1a), a droplet-collision extraction configuration with three gas channels, which was also used for dual sprays simultaneously with high DC powers (Figure S1b), a dual tip-mixing extraction configuration with two gas channels (Figure S1c), and a dual droplet-collision extraction configuration with four gas channels (Figure S1d).

### Fabrication of MCMCI

MCMCI was fabricated by polydimethylsiloxane (PDMS) utilizing standard multilayer soft lithography techniques as we have reported for fabricating the microfluidic chip-based sonic spray ionization^9^. The completely detailed fabrication process is shown in S1, ESI†. Five layers of MCMCI are illustrated in Fig. 1a. The top half chip included convex layer, gas channel layer, and liquid channel layer, whereas the bottom half chip contained gas channel layer and convex layer. Gas channel layers and convex layers were the same in the top and bottom half chips to form a symmetrical structure.

### MCMCI for extraction

For tip-mixing and droplet-collision extraction experiments, high voltage (5 kV) was applied on the extractive solvent, which was a mixture of methanol/water/acetic acid (70: 20: 10). The samples were urine (diluted five times by purified water) and purified water spiked separately with rhodamine B. Rhodamine B served as the standard determinant, and the urine served as the real biological solvent background. The pressures on the liquid channels were all 300 mbar, whereas the pressure was all approximately 3.5 bar on the gas channel inlet; such gas pressure was the optimized value for single channel ESI according to our previous work^1,9^, and thus was selected for each channel in our multiple channel experiments. Under such conditions, the flow rate was approximately 5.6 µL/min, which was similar to the flow rate of 5–10 µL/min that is usually used in EESI.

### MCMCI for dual sprays with high DC powers

As shown in Fig. 1d, apart from the droplet-collision extraction, the MCMCI with three gas channels was also developed for and dual sprays with high DC voltages applying simultaneously on two liquid channels, where the two liquids sprayed separately. In the experiments of dual sprays with high DC powers on, two liquid channels, where high voltages (5 kV) were all applied on, introduced two different samples. The pressures on the liquid channels were all 300 mbar, whereas the pressure was all approximately 3.5 bar on the gas channel inlet.

### MCMCI for dual EESI

Dual EESI integrates the functions of signle EESI and dual sprays with high DC powers. Thus, the experimental conditions of dual EESI combined the conditions of the above two functions. High voltages (5 kV) were applied on the two extracted solvents, which were distributed at the two sides of the sample liquid channel. The pressures on the liquid channels were also 300 mbar, whereas the pressure was all approximately 3.5 bar on the gas channel inlet.

### The coupling of MCMCI with mass spectrometry

The coupling of MCMCI with a thermo LCQ MS is shown in Fig. 1b. The optimized distance between MS inlet and microfluidic chip was approximately 6 mm, which was adjusted by a multi-dimensional manipulator; such optimized distance was determined to be around 5mm-6mm for our expermiment platform for coupling microfluidic ESI with MS in our previous work^35^. Compared with macro EESI, MCMCI was much easy to couple with MS because the angle between two sprayers was not required to optimize.

### Data Availability

All data generated or analysed during this study are included in this published article (and its Supplementary Information files).

## Electronic supplementary material

supplementary information
