# Supplementary material for: Multi-channel microfluidic chip coupling with mass spectrometry for simultaneous electro-sprays and extraction
Source: Sci Rep. 2017 Dec 12;7:17389. doi: 10.1038/s41598-017-17764-6 (PMC5727197; doi:10.1038/s41598-017-17764-6)
Supplement: Supplementary file 1 — supplementary information [file 41598_2017_17764_MOESM1_ESM.pdf]

Scientific Reports

Supplementary information

Multi-channel microfluidic chip coupling with mass spectrometry for simultaneous electro-sprays and extraction

Cilong Yu[1,2,4,+], Fei Tang[4,+], Xiang Qian[2,*], Yan Chen[3], Quan Yu[2], Kai Ni[2], and Xiaohao Wang[2,4,*]

[1]College of Mechatronics and Control Engineering, Shenzhen University, Shenzhen, 518060, China
[2]Division of Advanced Manufacturing, Graduate School at Shenzhen, Tsinghua University, Shenzhen 518055, China
[3]Shenzhen Institutes of Advanced Technology, Chinese Academy of Sciences, Shenzhen 518055, China
[4]The State Key Laboratory of Precision Measurement Technology and Instruments, Department of Precision Instrument, Tsinghua University, Beijing 100084, China
Corresponding: qian.xiang@sz.tsinghua.edu.cn; wang.xiaohao@sz.tsinghua.edu.cn
+these authors contributed equally to this work

Contents:

S1. Fabrication process of microfluidic chip
S2. Figures

Figure S1. Schematic of the microfluidic chip-based multi-channel ionization (MCMCI).

Figure S2. MS spectrum of 1 µM Rhodamine B in methanol and water(1:1). The commercial electrospray ionization was utilized with 5 KV high voltage.

Figure S3. (a) Long term stability of purified water spiked with 6 µM Rhodamine B. (b) MS spectrum of the purified water spiked with 6 µM Rhodamine B. The MCMCI with two gas channels was utilized.

Figure S4. MS spectrum of 1 µM Rhodamine B in urine (a) undiluted urine; (b) diluted urine by five times with methanol and water (1:1). The commercial electrospray ionization was utilized with 5 KV high voltage.

Figure S5. The configuration of macro EESI coupling with MS.

Figure S6. MS spectrum of the purified water spiked with 6 µM Rhodamine B. The MCMCI with three gas channels was utilized.

Figure S7. MCMCI with four gas channels and three liquid channels for dual droplet-collision EESI.

Figure S8. The detailed schematic of the whole experimental platform.

S1. Fabrication process of microfluidic chip

The microfluidic chip was fabricated using standard multilayer soft lithography techniques. The lithography process was as the following steps:

(1). A 3-inch silicon wafer template was treated in oxygen plasma (PDC-M, Chengdu Mingheng Science & Technology Co., Ltd, Chengdu, China) to prevent SU-8 photoresist from spalling;

(2). The negative photoresist (SU-8 2025) was then poured on the silicon wafer for spinning and soft-baking;

(3). The photoresist was exposed via photo mask A. This photo mask served as the liquid channel layer and provided an orifice and a channel for the liquid samples; The width and depth of liquid channel were 30 and 25 μm respectively;

(4). Putting this SU-8 master mold on thermostatic platform for post-baking;

(5). The second layer of negative photoresist (SU-8 2100) was applied at the top of the liquid channel layer for spinning and soft-baking, without developing uncross-linked photoresist;

(6). Photo mask B, which was aligned with the liquid channel layer by a UV aligner, was placed on the second layer photoresist for exposure. This second layer served as the gas channel layer with the channels and orifice for the gas flow; The width and depth of gas channel were about 30 and 200 μm respectively.

(7). Putting this SU-8 master mold on thermostatic platform for post-baking again;

(8). The third layer of negative photoresist (SU-8 2100) was applied at the top of the gas channel layer for spinning and soft-baking. The development of uncross-linked photoresist was also avoided;

(9). Photo mask C, which was aligned with the gas channel layer by a UV aligner, was placed on the third layer photoresist for exposure. This third layer served as the convex layer with the convex plate edge for cutting;

(10). Putting this SU-8 master mold on thermostatic platform for post-baking;

(11). The SU-8 photoresist layers were developed in propylene glycol methyl ether acetate;

(12). Then, this SU-8 master mold was placed on the thermostatic platform for hard-baking.

This SU-8 master mold served as the top layer of our micro-channel structure. Another SU-8 master mold with patterns of photo masks B and C only was prepared on another 3-inch silicon wafer template for the bottom PDMS micro-channel half-devices. This SU-8 master mold only had gas channel and convex layers; hence, the fabrication process was step (5) – (12).

The fabrication procedure of microfluidic chip using polydimethylsiloxane (PDMS) was as follows:

(1). The SU-8 master molds were modified with vapor-phase chlorotrimethylsilane to assist the release of PDMS membranes;

(2). PDMS base monomer and curing agent were mixed at 10:1 and 5:1 weight ratios, and then the mixtures were poured on the top and bottom SU-8 master molds separately;

(3). The two half-pieces were degassed under vacuum for about 20 minutes;

(4). The two half-pieces were then cured in an oven at 80 °C for 0.5 h;

(5). Two PDMS slabs were peeled off from the two master molds, and the inlet holes were drilled at the top by using a punch (tip diameter of 0.75 mm);

(6). Cutting off the excess PDMS. In general, considerable attention should be paid to remove the excess PDMS along the nozzle tips with a razor blade. However, in this study, a razor blade was only required to cut off the excess PDMS along the convex plate edge,;

(7). Both PDMS slabs were treated in oxygen plasma (PDC-M, Chengdu Mingheng Science & Technology Co., Ltd, Chengdu, China), and then bonded together by using an xyz-manipulator (Beijing

Optical Century Instrument Co., Ltd., Beijing, China);

(8). the PDMS microfluidic chip was cured at 80 °C for 48 h to enhance the bonding strength and eliminate the MS background from PDMS.

S2. Figures

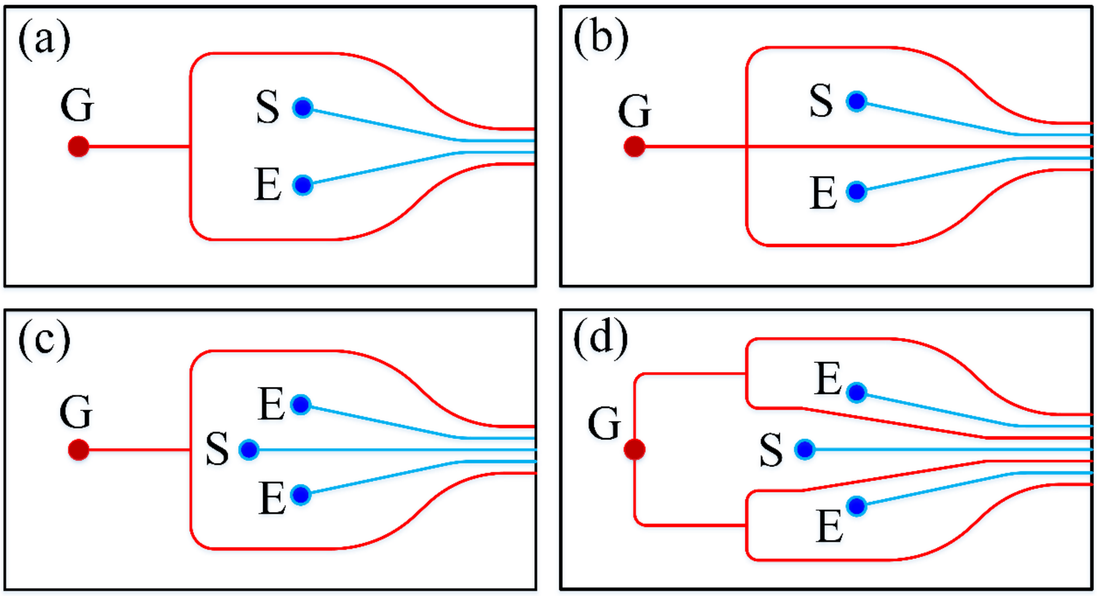

Figure S1. Schematic of the microfluidic chip-based multi-channel ionization (MCMCI). (a) MCMCI with two gas channels for tip-mixing EESI. (b) MCMCI with three gas channels for dual sprays with high DC powers simultaneously. (c) MCMCI with two gas channels for dual tip-mixing EESI. (d) MCMCI with four gas channels for dual droplet-collision EESI. All the red lines represented the gas channels and the blue lines represented the liquid channels. All the circles were the inlets of channels. G: gas channel inlet; S: sample solution inlet; E: extracted solvent inlet.

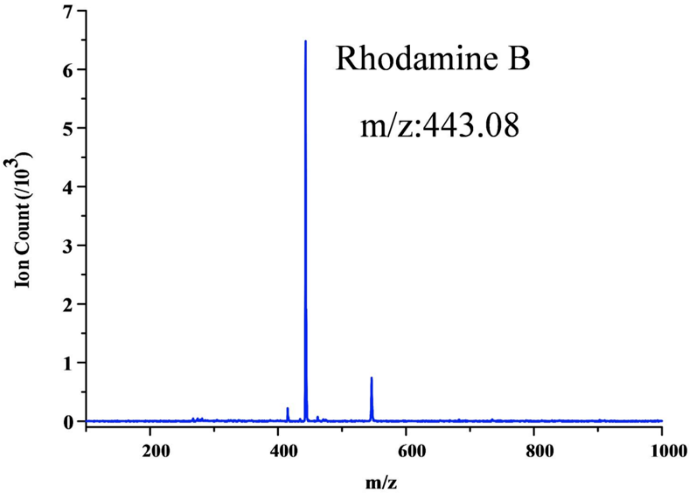

Figure S2. MS spectrum of 1 μM Rhodamine B in methanol and water(1:1). The commercial electrospray ionization was utilized with 5 KV high voltage.

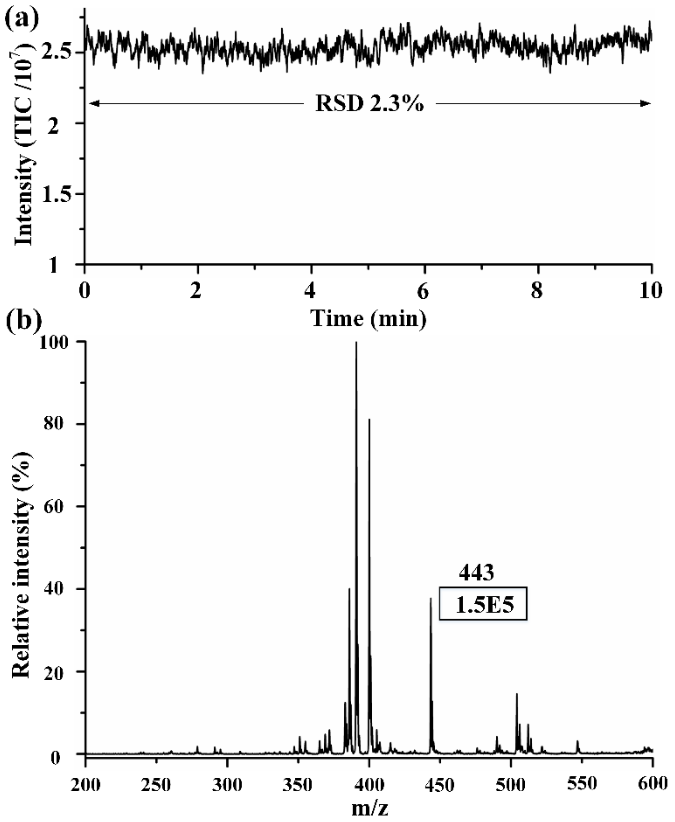

Figure S3. (a) Long term stability of purified water spiked with 6 μM Rhodamine B, extractive solvent was a mixture of methanol/water/acetic acid (70 : 20 : 10), high voltage (5 kV) was applied on extractive solvent. (b) MS spectrum of the purified water spiked with 6 μM Rhodamine B. The MCMCI with two gas channels was utilized. This was in the tip-mixing extraction mode.

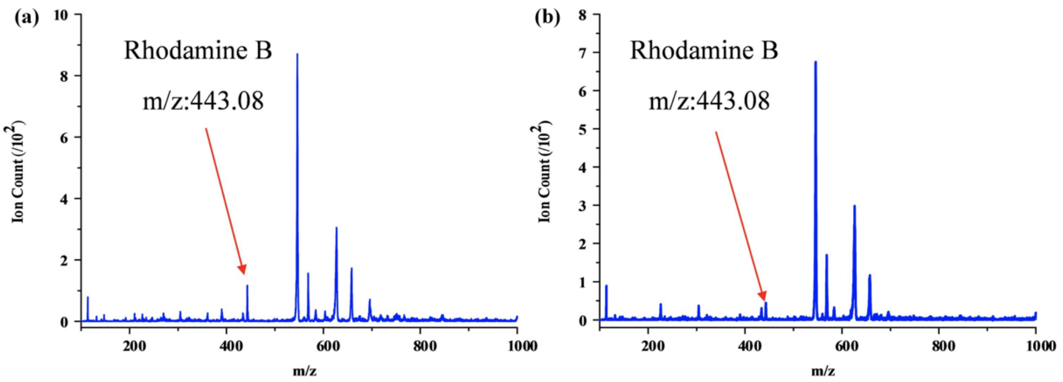

Figure S4. MS spectrum of 1 μM Rhodamine B in urine (a) undiluted urine; (b) diluted urine by five times with methanol and water (1:1). The commercial electrospray ionization was utilized with 5 KV high voltage.

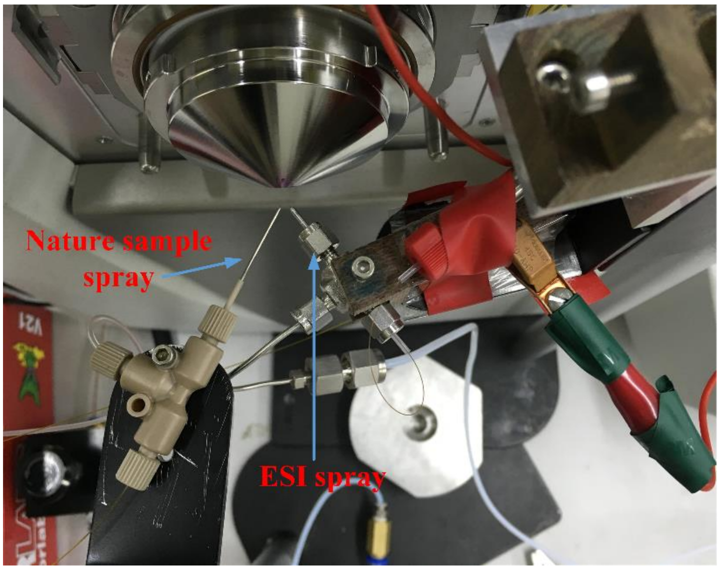

Figure S5. The configuration of macro EESI coupling with MS.

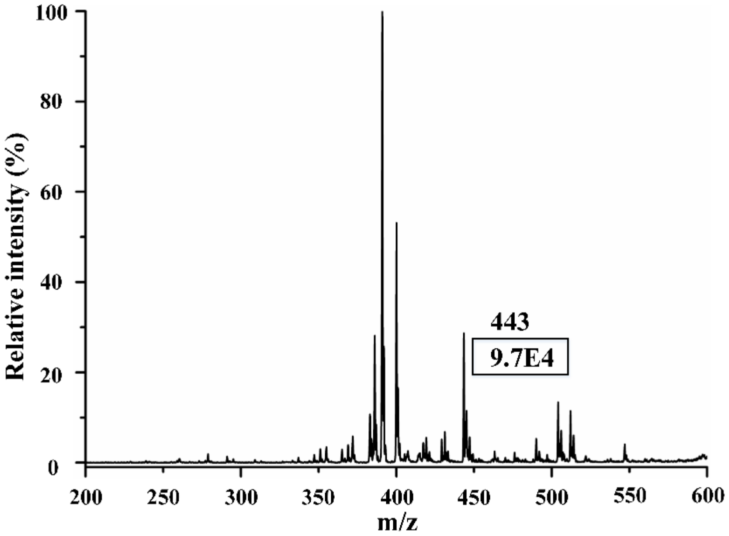

Figure S6. MS spectrum of the purified water spiked with 6 μM Rhodamine B. Extractive solvent was a mixture of methanol/water/acetic acid (70 : 20 : 10), high voltage (5 kV) was applied on extractive solvent. The MCMCI with three gas channels was utilized. This was in the droplet-collision extraction mode.

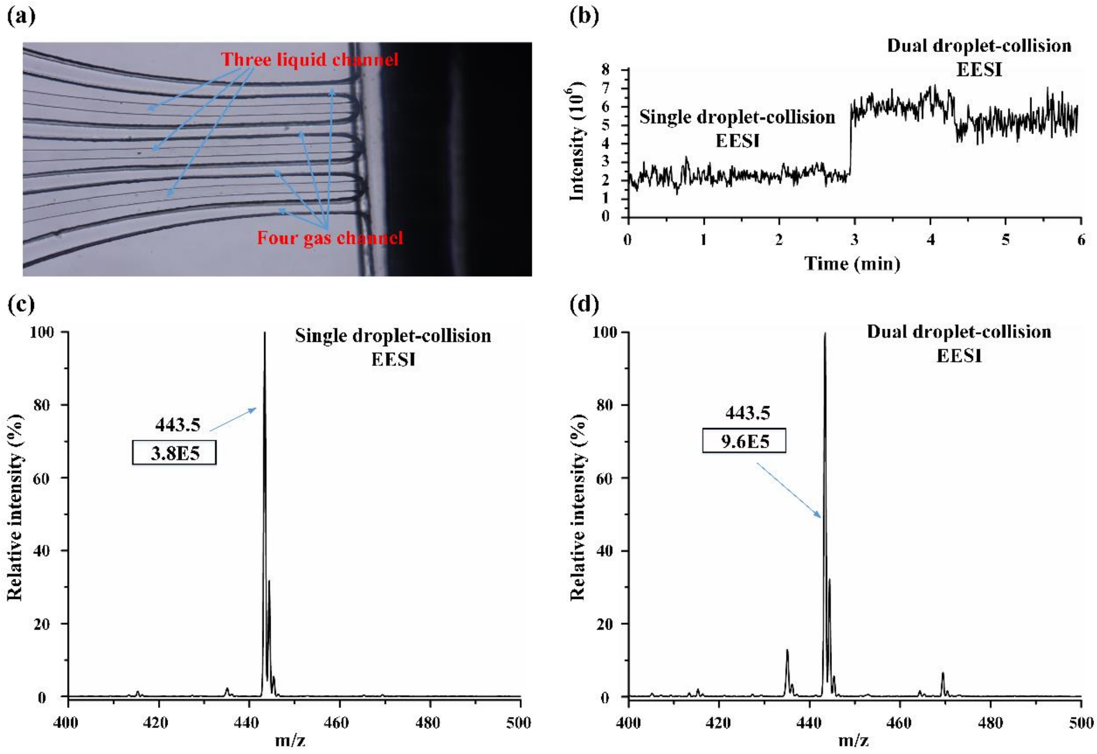

Figure S7. (a) The structure of MCMCI with four gas channels and three liquid channels for dual droplet-collision EESI. (b) TIC intensity of 5 μM rhodamine B (extracted mass range from 443 to 444). In the first 3 minute, only one extracted channel sprayed with sample channel (*i.e.*, the single droplet-collision EESI); in the last 3 minute, two extracted channels sprayed with sample channel (*i.e.*, the dual droplet-collision EESI). (c) MS spectrum of 5 μM rhodamine B in the single droplet-collision EESI mode. (d) MS spectrum of 5 μM rhodamine B in the dual droplet-collision EESI mode.

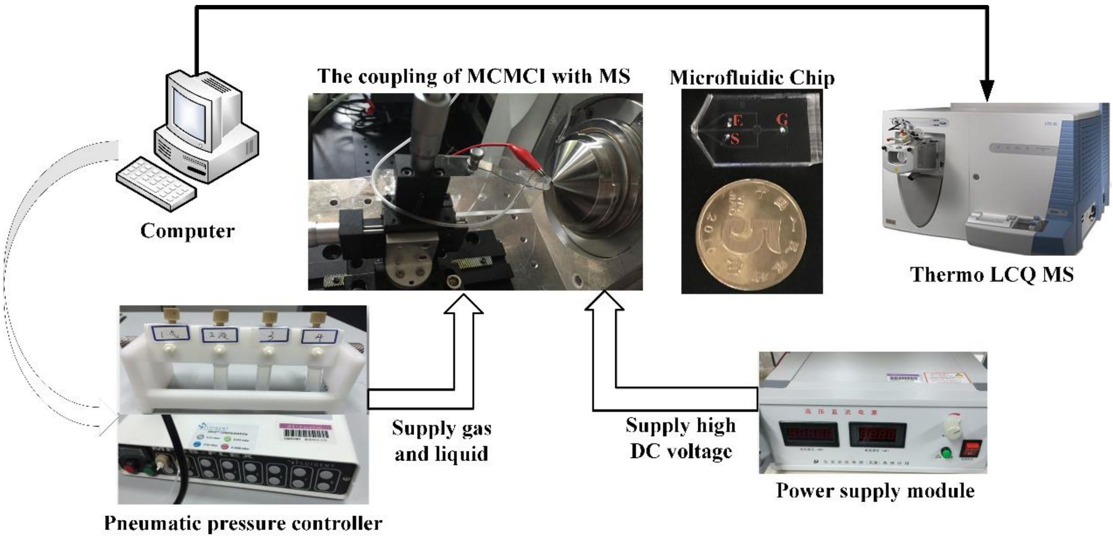

Figure S8. The detailed schematic of the whole experimental platform. The gas and liquid were introduced by a pneumatic pressure controller. The high DC voltage supplied by a power supply module was applied on the stainless steel tubes, which were embedded in the reservoirs in the MCMCI. The MCMCI was held by a laboratory-built platform and the distance between the microfluidic chip emitter and the MS inlet orifice was adjusted by a xyz-manipulator. The Thermo LCQ MS and pneumatic pressure controller were controlled by

computer. The characters ‘G’, ‘S’, ‘E’ on the chip are the same as figure S1 as, G: gas channel inlet; S: sample solution inlet; E: extracted solvent inlet.
